# Functional neurological disorder on YouTube: how reliable is the information?

**DOI:** 10.3389/fpubh.2026.1802156

**Published:** 2026-04-07

**Authors:** Murat Alpua, Tahir Kurtuluş Yoldaş

**Affiliations:** 1 Department of Neurology, Faculty of Medicine, Kırıkkale University, Kırıkkale, Türkiye; 2 Department of Neurology, Faculty of Medicine, Ankara Yıldırım Beyazıt University, Ankara, Türkiye

**Keywords:** digital health literacy, functional neurological disorder, health information quality, patient education, YouTube

## Abstract

**Background:**

Functional Neurological Disorder (FND) is a common and often misunderstood condition characterized by neurological symptoms such as limb weakness, movement disorders, sensory disturbances, and non-epileptic seizures that are not explained by structural neurological disease. Patients increasingly seek information through digital platforms such as YouTube; however, the reliability and educational value of such content remain uncertain.

**Objective:**

This study aimed to systematically evaluate the quality, reliability, and educational value of English-language YouTube videos on FND using standardized assessment tools.

**Methods:**

A cross-sectional analysis was conducted on the 50 most viewed videos retrieved with relevant keywords. Video characteristics and engagement metrics were recorded. Quality was assessed using the Global Quality Scale (GQS), reliability with the modified DISCERN (mDISCERN), and health information standards with JAMA benchmark criteria. User interaction was measured via the Video Power Index (VPI). Statistical analyses included Spearman correlation, Kruskal–Wallis and Mann–Whitney U tests, with effect sizes reported. Inter-rater reliability was evaluated using ICC and weighted Cohen’s kappa.

**Results:**

The mean GQS, mDISCERN, and JAMA scores were 3.27, 3.23, and 2.38, respectively, indicating moderate overall quality but suboptimal adherence to health information standards. Producer type did not significantly affect quality scores (*p* > 0.05), though VPI differed across groups (*p* = 0.022), with health information channels showing higher engagement. VPI showed strong correlations with both view count and like count. Engagement metrics demonstrated limited association with information quality indicators. Inter-rater reliability was excellent across all instruments (ICC range: 0.882–0.944).

**Conclusion:**

YouTube hosts a substantial amount of FND-related content; however, overall quality and reliability are inconsistent. Engagement metrics do not reliably reflect informational accuracy. Given the stigma and complexity of FND, reliance on unregulated online content may hinder patient understanding and management. Greater involvement of clinicians and professional organizations in producing evidence-based, patient-centered digital resources is warranted to improve health literacy and outcomes.

## Introduction

Functional Neurological Disorder (FND) is a common and clinically significant disorder characterized by motor, sensory, or cognitive neurological symptoms without a structural neurological pathology. In current classification, FND is considered a spectrum encompassing heterogeneous clinical presentations such as functional weakness, tremor, dystonia, gait disturbance, and functional seizures (1). Neurobiological and clinical studies show that FND is not merely a psychogenic condition; it is a complex disease associated with abnormal brain networks, impaired sensory-motor integration, and expectancy mechanisms (1, 2).

FND is frequently seen among patients presenting to neurology clinics and constitutes a significant portion of new patient referrals (3). Despite this, the disease is often misunderstood by both patients and healthcare professionals, and the diagnostic process can be delayed. This can negatively impact the prognosis and make it difficult for patients to access appropriate treatment approaches (4). Therefore, in FND, patient education and accurate information are considered a fundamental component in terms of diagnosis acceptance and treatment adherence (1, 4).

Today, patients increasingly obtain health-related information from the internet and social media platforms. YouTube, in particular, has become one of the most widespread sources of health information due to its visual and auditory presentation, easy accessibility, and large user base (5, 6). However, health content on YouTube is largely unregulated, and the scientific accuracy, reliability, and educational value of videos can vary significantly (5–7).

Numerous studies in the field of neurology have shown that YouTube videos often contain incomplete, superficial, or misleading information for various neurological diseases such as Parkinson’s disease, and multiple sclerosis (8, 9). It has also been shown that popularity indicators such as view counts and likes are not always parallel to content quality (5, 7). This is particularly important for FND, a disease prone to misunderstanding and stigma. Incorrect or reductionist information can lead patients to refuse diagnosis, resort to inappropriate treatments, and damage the physician-patient relationship.

Although numerous studies in the literature evaluate the quality and reliability of YouTube content, there is no specific study that systematically examines the quality and reliability of YouTube videos aimed at providing information about Functional Neurological Disorder (FND). This situation points to a significant knowledge gap in terms of digital health literacy in the field of FND.

The aim of this study is to examine the quality, reliability, and educational value of YouTube videos published in English and aiming to provide information about Functional Neurological Disorder using standardized assessment scales, and to evaluate whether YouTube is a reliable source of information for FND.

## Materials and methods

### Search strategy and video selection

The YouTube search was performed using the standard YouTube interface without a logged-in account and using Google Chrome in incognito mode to minimize personalization effects. Searches were conducted from Turkey on December 23, 2025. Results were sorted by view count, and the first 61 videos retrieved using the predefined keywords. YouTube videos were searched using a combined free-text query including the terms “Functional Neurological Disorder,” “Functional neurological symptom disorder,” “FND,” and “Conversion disorder.” These keywords were entered together in a single search to reflect real-world user behavior. Boolean operators were not explicitly used, as YouTube does not function as a traditional database and applies its own algorithm to retrieve results. Only English-language videos were eligible for inclusion. Non-informative and short videos were excluded from the evaluation. Videos shorter than 30 s were classified as “short videos” and were excluded because such content typically corresponds to brief clips or YouTube Shorts that lack sufficient educational depth. “Non-informative videos” were defined as videos that did not provide explanatory or educational information about Functional Neurological Disorder, such as promotional material, unrelated commentary, personal reactions without discussion of the disorder, or clips lacking information on symptoms, diagnosis, mechanisms, or treatment. These predefined criteria were applied during the screening process to reduce selection bias. After applying the inclusion and exclusion criteria, the 50 most viewed eligible videos were included in the final analysis. Two neurologists (M. A. and T. K. Y.) independently reviewed each video.

### Data extraction and video characteristics

Because the analyzed material was publicly available, included no identifiable personal information, and did not involve human subjects, institutional ethics approval was not necessary. For each video, we extracted data on view count, duration, likes and dislikes, uploader category, upload year, video age, interval between upload and assessment, number of comments, content type, country of origin, symptom description, inclusion of etiology/diagnosis/treatment information, target audience, and advertising presence. Because YouTube no longer publicly displays dislike counts, dislike data were obtained using the Return YouTube Dislike browser extension, which estimates dislike counts based on archived platform data and user interaction patterns. Producers were classified into six categories: patients (individuals describing personal experiences), physicians, health/medical information channels (channels predominantly focused on health-related topics), associations (professional bodies or patient organizations), hospitals/healthcare centers, and others (e.g., television programs with health content, psychologists, and creators not fitting the predefined groups).

### Quality and reliability assessment (quality indicators)

For a structured and unbiased appraisal, we relied on established evaluation instruments widely used and validated in previous studies evaluating online health information (10–12). The Global Quality Scale (GQS) provided an overall quality rating from 1 to 5, with higher values indicating superior quality and greater usefulness to patients (10). Adherence to health information standards was evaluated according to the JAMA benchmark criteria, covering authorship, attribution, currency, and disclosure. Each domain was rated dichotomously (0 or 1), and total scores were categorized as adequate (4), partially adequate (2–3), or inadequate (0–1) (11). Reliability and consistency were examined using the five-item modified DISCERN (mDISCERN) tool (0–5 points), which evaluates clarity of objectives, credibility of sources, balance, availability of supplementary resources, and acknowledgment of uncertainty (12).

### Engagement metrics (engagement indicators)

User interaction was evaluated using the Video Power Index (VPI), a composite metric that reflects both the popularity and engagement level of a video. VPI is derived from the like rate and view rate, where the view rate represents the average number of daily views since upload, and the like rate indicates the proportion of positive reactions among total user feedback. This metric has been widely used in previous studies to assess the visibility and audience interaction of health-related videos on YouTube (13). In the context of the present study, VPI was used to explore whether viewer engagement is associated with the quality and reliability of the information presented.

### Statistical analysis

Data analysis was conducted in IBM SPSS Statistics version 23. Continuous variables were described using mean ± standard deviation, median, and range, whereas categorical data were expressed as counts and percentages. The distribution of continuous variables was assessed using visual (histograms, Q–Q plots) and analytical methods. As most variables did not follow a normal distribution, non-parametric tests were applied. Spearman correlation was preferred due to non-normal data distribution. Associations between quality measures (GQS, mDISCERN, JAMA) and engagement indicators were explored with Spearman correlation. Differences between groups were tested using the Mann–Whitney U test or Kruskal–Wallis test as appropriate, and effect sizes were reported as rank-biserial correlation and eta-squared. Inter-rater agreement was assessed using the Intraclass Correlation Coefficient (ICC)(two-way random, absolute agreement, single measure) and quadratic weighted Cohen’s kappa. Statistical significance was set at *p* < 0.05. Because multiple correlation analyses were performed, the risk of Type I error (false-positive findings) increases with repeated testing. Therefore, a Bonferroni correction was applied to control for multiple comparisons, and the significance threshold was adjusted accordingly (*p* < 0.0042).

## Results

Descriptive statistics are summarized in Table 1. The mean view count was 41,494, with a mean video duration of 829 s. Videos received a mean of 595 likes and 89 comments. The mean time since upload was 4.8 years. Mean quality scores were 3.27 for GQS, 3.23 for mDISCERN, and 2.38 for JAMA, while the mean Video Power Index (VPI) was 26.12.

**Table 1:** Characteristics of all videos.

Variable	Mean	Median	Standard deviation	Minimum	Maximum
View count	41494.4	14055.5	53118.07	4,381	255,228
Duration (s)	828.98	344	1086.12	35	5,022
Like count	594.56	240	891.13	27	4,100
Dislike count	18.42	5.5	36.14	0	218
Comment count	88.69	35.5	142.93	0	595
Age (years)	4.8	4	3.09	0	12
Days since upload	1948.16	1,637	1142.12	256	4,732
GQS	3.27	3	0.85	2	5
mDISCERN	3.23	3	0.83	2	5
JAMA	2.38	2	0.81	1	4
VPI	26.12	11.5	36.97	1	167

Producer distribution is presented in Table 2, with associations being the most common source (30%), followed by health information channels (24%) and hospitals or health centers (20%).

**Table 2:** Distribution of videos by producers.

Label	*n*	%
Patient	5	10
Physician	3	6
Health information channel	12	24
Association	15	30
Hospital	10	20
Other	5	10

Comparisons of video quality scores across producer groups are shown in Table 3. No significant differences were observed between producer types for GQS, mDISCERN, or JAMA scores (all *p* > 0.05). However, VPI differed significantly across groups (*p* = 0.022), with health information channels demonstrating higher VPI values compared to associations.

**Table 3:** Comparison of video quality scores across producer groups.

Producer	Statistic	GQS	mDISCERN	JAMA	VPI
Patient	Mean ± SD	3.1 ± 0.74	3.1 ± 0.74	2.1 ± 0.74	40.2 ± 55.1
	Median [25th–75th percentile]	3 [2.5–3.75]	3 [2.5–3.75]	2 [1.5–2.75]	8 [3–93.5]
Medical doctor	Mean ± SD	4.0 ± 0.50	4.0 ± 0.50	3.0 ± 0.50	5.33 ± 4.9
	Median [25th–75th percentile]	4 [3.5–4.5]	4 [3.5–4.5]	3 [2.5–3.5]	3 [2–11]
Health information channel	Mean ± SD	3.42 ± 0.93	3.33 ± 1.00	2.67 ± 0.78	46.8 ± 52.6
	Median [25th–75th percentile]	3.75 [2.63–4]	3.75 [2.13–4]	3 [2–3]	20 [6.5–81.8]
Association	Mean ± SD	3.07 ± 0.84	3.07 ± 0.84	2.2 ± 0.77	7.21 ± 8.38
	Median [25th–75th percentile]	3 [2.5–4]	3 [2.5–4]	2 [2–3]	4 [2–8.25]
Hospital/Health center	Mean ± SD	3.15 ± 1.00	3.05 ± 0.83	2.2 ± 1.03	20.3 ± 12.18
	Median [25th–75th percentile]	3 [2–4]	3 [2–4]	2 [1–3]	20 [11–24.75]
Others	Mean ± SD	3.5 ± 0.5	3.5 ± 0.5	2.5 ± 0.5	43.0 ± 42.96
	Median [25th–75th percentile]	3.5 [3–4]	3.5 [3–4]	2.5 [2–3]	31.5 [9.25–88.25]
*p*		0.402	0.424	0.268	0.022¥

¥Association-health information channel different.

Correlation analyses are presented in Table 4. Like count was positively correlated with GQS (*r* = 0.326, *p* = 0.024) and JAMA scores (*r* = 0.405, *p* = 0.004). VPI showed strong positive correlations with both view count (*r* = 0.821, *p* < 0.001) and like count (*r* = 0.819, *p* < 0.001). A moderate positive correlation was also observed between VPI and JAMA scores (*r* = 0.371, *p* = 0.009). After applying Bonferroni correction for multiple testing (adjusted significance threshold *p* < 0.0042), only the correlations between VPI and view count, VPI and like count, and like count and view count remained statistically significant.

**Table 4:** Correlations between the number of views and likes and the evaluation tools.

Variables	Correlation coefficient	%95 CI	*p*
GQS–view count	0.149	−0.135 – 0.410	0.303
GQS–like count	0.326	0.046–0.558	0.024
mDISCERN–view count	0.087	−0.196 – 0.357	0.548
mDISCERN–like count	0.278	−0.006 – 0.521	0.055
JAMA–view count	0.241	−0.040 – 0.487	0.091
JAMA–like count	0.405	0.137–0.618	0.004
VPI–view count	0.821	0.700–0.896	<0.001
VPI–like count	0.819	0.697–0.895	<0.001
VPI–mDISCERN	0.228	−0.060 – 0.481	0.120
VPI–GQS	0.282	−0.002 – 0.524	0.052
VPI–JAMA	0.371	0.098–0.593	0.009
Like count–view count	0.864	0.768–0.922	<0.001

Bonferroni correction for multiple testing was applied (adjusted significance threshold *p* < 0.0042).

Inter-rater reliability was excellent for all assessment tools (Table 5), with intraclass correlation coefficients of 0.885 for GQS, 0.882 for mDISCERN, and 0.944 for JAMA (all *p* < 0.001).

**Table 5:** Inter-rater reliability.

Assessment tool	ICC (Intraclass correlation coefficient)	*p*
GQS	0.885	<0.001
mDISCERN	0.882	<0.001
JAMA	0.944	<0.001

## Discussion

In this study, we systematically evaluated the quality, reliability, and educational value of the most viewed English-language YouTube videos providing information on Functional Neurological Disorder (FND). To the best of our knowledge, limited research has examined the quality and reliability of YouTube videos specifically related to Functional Neurological Disorder (FND). Our findings suggest that while YouTube contains a moderate amount of potentially useful information on FND, overall content quality and reliability remain variable and frequently suboptimal.

The mean GQS and modified DISCERN scores in our sample indicated a moderate level of overall quality and reliability. However, the relatively low mean JAMA score highlights deficiencies in fundamental health information standards, particularly regarding authorship transparency, attribution of sources, and disclosure. These results align with earlier investigations of YouTube content addressing neurological conditions including Parkinson’s disease, multiple sclerosis, and epilepsy, where videos often lacked clear sourcing and balanced discussion despite appearing professionally produced or widely viewed (14–17).

The present findings are also consistent with studies conducted in other medical fields beyond neurology. Previous research evaluating YouTube content on topics such as cancer, and surgical procedures has similarly demonstrated that online health information is often heterogeneous in quality and frequently lacks adherence to established scientific standards (11, 12). These studies consistently report that engagement metrics, including views and likes, do not reliably reflect the accuracy or educational value of the content. This broader body of evidence suggests that the limitations of YouTube as a health information source represent a cross-disciplinary issue rather than a problem specific to neurological disorders.

An important finding of our study is the lack of significant differences in quality scores across different producer categories, including physicians, hospitals, associations, and health information channels. This suggests that professional affiliation alone does not guarantee high-quality or reliable content. Similar observations have been reported in a prior review, emphasizing that medical authority is not consistently translated into better digital health communication (18). In contrast, engagement metrics differed significantly between producer groups, with health information channels achieving higher VPI values despite not demonstrating superior quality scores. This discrepancy underscores the well-documented mismatch between popularity and informational quality on YouTube.

Correlation analyses further support this observation, although most associations between engagement metrics and information quality indicators did not remain statistically significant after Bonferroni correction for multiple testing. Only the correlations between VPI and view count, VPI and like count, and between like count and view count remained statistically significant. This finding indicates that engagement metrics were strongly interrelated, whereas their association with information quality indicators was limited. Presentation style, storytelling, emotional appeal, and algorithmic promotion may play a substantial role in visibility and interaction, potentially amplifying videos with simplified or incomplete explanations (19).

Functional Neurological Disorder has historically been associated with substantial stigma and misunderstanding, partly due to its earlier conceptualization as a purely psychogenic condition. Although contemporary research highlights neurobiological mechanisms such as altered brain network functioning and abnormal sensory–motor integration, public understanding often continues to reflect older psychological interpretations. In digital media environments such as YouTube, this tension may influence how the disorder is framed and communicated. Oversimplified explanations that emphasize either purely psychological or purely neurological interpretations may fail to capture the current biopsychosocial understanding of FND. Such representations may affect patients’ acceptance of the diagnosis, trust in medical explanations, and willingness to engage in recommended treatments. Therefore, the accuracy and balance of online information may be particularly important for FND compared with many other neurological conditions.

Previous studies have demonstrated that patient understanding and validation of the diagnosis are critical determinants of prognosis in FND (20, 21). Therefore, unreliable online information may indirectly contribute to poorer clinical outcomes.

From a clinical perspective, these findings highlight the importance of clinicians being aware of the type and quality of information patients may encounter online. Many patients with Functional Neurological Disorder seek explanations for their symptoms through digital platforms before or after medical consultations. Inaccurate or overly simplified representations of FND on widely viewed platforms such as YouTube may influence patients’ understanding of the diagnosis and their acceptance of recommended treatments. Therefore, clinicians may benefit from actively guiding patients toward reliable educational resources and evidence-based digital content.

Nonetheless, this study has several limitations. First, the analysis was restricted to English-language videos and the 50 most viewed results, which may limit generalizability. Second, YouTube content is dynamic, and video metrics may change over time. Third, Another limitation is the relatively small number of videos in certain producer categories, particularly the physician group. This imbalance may limit the statistical power of between-group comparisons and reduce the reliability of conclusions regarding differences between producer types. Therefore, the results related to producer categories should be interpreted cautiously. Finally, assessment tools such as GQS and DISCERN, while widely used, involve a degree of subjectivity.

Despite these limitations, our findings highlight a clear need for neurologists, professional organizations, and academic institutions to take a more active role in producing high-quality, patient-centered digital content on FND. Standardized, evidence-based videos that address symptoms, mechanisms, diagnosis, and treatment in a balanced and transparent manner may help counter misinformation and improve patient education.

## Conclusion

YouTube contains a substantial amount of content related to Functional Neurological Disorder; however, the overall quality and reliability of this information are inconsistent and frequently inadequate. Viewer engagement and popularity metrics do not reliably reflect informational accuracy or educational value. Given the complexity and stigma associated with FND, reliance on unregulated online content may pose risks for patient understanding and disease management. Clinicians should be aware of the limitations of YouTube as an information source and proactively guide patients toward reliable educational materials. Future efforts should focus on developing and promoting high-quality, evidence-based digital resources to improve health literacy and patient outcomes in FND.

## Data Availability

The raw data supporting the conclusions of this article will be made available by the authors, without undue reservation.
